# Upregulation of glycolytic enzyme PFKFB3 by deubiquitinase OTUD4 promotes cardiac fibrosis post myocardial infarction

**DOI:** 10.1007/s00109-023-02323-6

**Published:** 2023-05-10

**Authors:** Feizuo Wang, Xiaojian Yin, Yuan-Ming Fan, Xinyao Zhang, Chao Ma, Keke Jia, Wei Zhou, Zongxiang Tang, Lian-Wen Qi, Jia Li

**Affiliations:** 1 School of Medicine & Holistic Integrative Medicine, Nanjing University of Chinese Medicine, No. 138 Xianlin Avenue, Nanjing, 210023 Jiangsu China; 2 State Key Laboratory of Natural Medicines, School of Traditional Chinese Pharmacy, China Pharmaceutical University, No. 639 Longmian Road, Nanjing 210009 Jiangsu, China

**Keywords:** Cardiac fibrosis, Glycolysis, Metabolic reprogramming, OTUD4, PFKFB3

## Abstract

**Abstract:**

Metabolic dysregulations have emerged as a major mediator of cardiovascular disorders and fibrotic diseases. Metabolic reprogramming contributes a lot to cardiac fibroblast activation and cardiac fibrosis post-myocardial infarction (MI), yet the mechanism remains incompletely understood. Our work aimed to determine whether or not glycolytic reprogramming, regulated by phosphofructokinase-2/fructose-2,6-bisphosphatase 3 (PFKFB3), is a therapeutic target for alleviating post-MI cardiac fibrosis. Here, we showed that cardiac fibroblasts displayed cell energy phenotype toward augmented glycolysis in response to transforming growth factor-beta 1 (TGF-β1), evidenced by significant extracellular acidification rate (ECAR) increase and lactate accumulation. The expression of glycolytic enzyme PFKFB3, a master activator of glycolysis, was up-regulated in TGF-β1-treated cardiac fibroblasts and in cardiac fibroblasts of post-MI mice. Pharmacological inhibition of PFKFB3 by 3PO diminished TGF-β1-mediated profibrotic phenotypes, attenuated cardiac fibrosis, and preserved cardiac functions in post-MI mice. Meanwhile, the genetic inhibition of PFKFB3 decreased the cardiac fibroblast activation and reversed the differentiated phenotypes in vitro and in vivo. Mechanistically, we identified deubiquitinase OTUD4 as a new binding protein of PFKFB3, and their interaction blocked PFKFB3 degradation via OTUD4-mediated deubiquitylation. Taken together, this work characterized a key role for PFKFB3 in cardiac fibroblast activation and suggested that inhibiting PFKFB3-involved glycolysis is an alternative way to alleviate post-MI cardiac fibrosis.

**Key messages:**

- PFKFB3, a master activator of glycolysis, was highly expressed in ischemic cardiac fibroblasts to enhance cardiac fibrosis
- The deubiquitinase OTUD4 was identified as a new binding protein of PFKFB3
- TGF-β1 blunted the ubiquitination-mediated degradation of PFKFB3 via OTUD4-mediated deubiquitylation
- Blockade of PFKFB3 contributed to ameliorating ischemia-induced cardiac fibrosis

**Supplementary Information:**

The online version contains supplementary material available at 10.1007/s00109-023-02323-6.

## Introduction

Cardiac fibrosis is a crucial aspect of remodeling of the failing heart, characterized by abnormal proliferation of interstitial fibroblasts and excessive deposition of extracellular matrix (ECM). After the ischemic injury, the damaged cardiomyocytes are replaced by fibrotic scars due to limited regenerative capacity. During the repair process, cardiac fibroblasts are activated and exhibit proliferative and secretive characteristics to prevent the rupturing of the ventricular wall [1, 2]. Excessive and sustained forces of cardiac fibroblast activation, however, could increase left ventricle stiffness and decrease ventricular wall compliance, resulting in impaired cardiac output [2]. Given that fibroblasts act as the critical mediator cells concerning pathological reactive fibrosis, targeting the activated cardiac fibroblasts may represent a strategy to prevent ischemia-induced cardiac fibrosis and heart failure.

Metabolic alterations are implicated in the pathogenesis of various diseases, such as cancer and cardiovascular diseases [3, 4]. A hallmark feature of metabolic reprogramming is the enhancement of aerobic glycolysis [5]. In cancer cells, aerobic glycolysis can produce ATP faster, and the increased glycolytic flux also generates byproducts to fuel proliferation [6]. Increasing evidence indicates that Warburg effects also act as a crucial role in non-tumor disease, including cardiac hypertrophy and failing heart [7, 8]. Indeed, increased glucose uptake and lactate production are also observed in proliferating non-cancer cells [9]. In fibrosis, metabolic reprogramming is required for myofibroblast contractility and differentiation [10]. Glycolysis has been demonstrated to be essential for regulating transforming growth factor beta 1 (TGF-β1) secretion and its downstream signalings [11]. Glycolysis is also necessary for ECM production in terms of amino acid synthesis, collagen hydroxylation, and ECM secretion [11, 12]. When the heart suffers ischemia, glycolysis is enhanced to supply amounts of energy quickly [13].

Glycolysis is a multi‐step reaction that is controlled by several rate‐limiting glycolytic enzymes, including hexokinase (HK), phosphofructokinase (PFK), and pyruvate kinase (PK). Targeting these rate‐limiting glycolytic enzymes is an ideal strategy for limiting cardiac fibrosis. However, direct inhibition of these enzymes may result in complete blockade of glycolytic flux and thus cause unfavorable effects [14], since they are indispensable for the remodeling of the cardiac tissue after ischemic injury [15]. Phosphofructokinase-2/fructose-2,6-bisphosphatase 3 (PFKFB3) is a master activator of glycolysis, which synthesize fructose-2,6-bisphosphate (F2,6BP), an allosteric activator of PFK1 [16]. Among all four PFKFB bifunctional isoenzymes, PFKFB3 has a much higher kinase activity than bisphosphatase activity to shunt glucose toward glycolysis [17]. PFKFB3 blockade could inhibit glycolysis partially and transiently, indicating its therapeutic potential [18]. PFKFB3 is highly expressed in many cancer cells and proliferating non-cancer cells [19]. The PFKFB3 protein level is affected by synthesis and degradation [20–22]. Its protein stability is controlled by the ubiquitin-dependent proteasomal pathway [22]. The role of PFKFB3-involved glycolysis in cardiac fibroblast activation after ischemic damage remains to be investigated.

This work sought to characterize the role of PFKFB3 in post-MI cardiac fibrosis. We observed a noticeable increase of PFKFB3 and glycolysis in TGF-β1-stimulated cardiac fibroblasts in vitro and in the hearts of post-MI mice. Genetic silencing and pharmacological inhibition were employed to investigate the functions of PFKFB3 in cardiac fibrosis. Co-immunoprecipitation combined with mass spectrometry (Co-IP/MS) was performed to identify PFKFB3-binding proteins and to explore the underlying mechanisms.

## Materials and methods

For additional experimental procedures, see Supplemental Methods.

## Animals and experimental model

C57BL/6 J male mice (6–8 weeks) were purchased from the Laboratory Animal Center of GemPharmatech Co. Ltd. (Nanjing, China). The mice were subjected to permanent ligation of the left anterior descending coronary artery (LAD) as described previously [23]. After surgery, 3PO (35 mg/kg) or vehicle (DMSO) was administrated by intraperitoneal injection every other day for 4 weeks. For knockdown PFKFB3, mice received a single-bolus AAV9-*Pfkfb3* shRNA or AAV9-NC (Hanbio Biotechnology, Shanghai, China) via tail vein injection, which may show clear silencing efficacy 2–4 weeks after infusion. After confirmation of successful *pfkfb3* knockdown 3 weeks after AAV-shRNA injection, LAD ligation surgery was conducted. A cardiac function assay was conducted 4 weeks post-myocardial infarction (MI). Then the hearts were isolated for Masson’s trichrome staining, picrosirius red staining, immunohistochemistry examination, or protein extraction. The animal care and all experimental procedures were approved by the Animal Ethics Committee of the Nanjing University of Chinese Medicine and the Animal Ethics Committee of China Pharmaceutical University.

## Cell preparation and culture

Neonatal rat cardiac fibroblasts (NRCFs) were isolated from 1- to 2-day-old SD rats from B&K Universal Group Ltd (Shanghai, China) following a reported method [24]. The prepared fibroblasts were treated with TGF-β1 (10 ng/ml) with or without PFKFB3 inhibitor 3PO (20 μM) for 24 h. For silencing *Pfkfb3*, the cardiac fibroblasts were transfected with siRNA specific for rat *Pfkfb3* (Hanbio Biotechnology) using Lipofectamine 3000 (Invitrogen, USA). After transfection, cells were stimulated with TGF-β1 (10 ng/ml) for 24 h.

## Cell energy phenotype test

NRCFs were plated in Seahorse XF Cell Culture Microplate and treated with or without TGF-β1 for 24 h. The extracellular acidification rate (ECAR) and mitochondrial oxygen consumption rate (OCR) of NRCFs were assayed by Agilent Seahorse XF Cell Energy Phenotype Test Kit (Seahorse Bioscience Inc., North Billerica, USA).

## Western blot assay

After indicated treatment, NRCFs were lysed. Equivalent amounts of protein were separated by SDS-PAGE and transferred onto PVDF membranes. After blocking, the membranes were incubated with primary antibodies, followed by incubation with HRP-conjugated secondary antibodies. The relative expression level of the target protein was normalized to housekeeping gene α-Tubulin content. The blots were quantified by ImageProPlus 6.0 software. The information for antibodies was shown in the Supplemental Methods.

## Co-immunoprecipitation assay

After being treated with TGF-β1 for 24 h, NRCFs were lysed by Pierce IP Lysis Buffer (Thermo Fisher Scientific). After centrifugation, part of the supernatant was taken out as input. The remaining supernatant was incubated overnight with either PFKFB3-specific polyclonal antibody (Proteintech, 13763–1-AP) or OTUD4 antibody (Novus Biologicals, NBP1-36976). Afterward, the lysates were treated with Pierce Protein A/G Magnetic Beads (Thermo Fisher Scientific). Bead-bound proteins were resolved by SDS-PAGE and detected by immunoblotting.

## Immunofluorescence

NRCFs were co-transfected with Flag-PRKFB3 and HA-OTUD4 plasmids (Hanbio Biotechnology). After being treated with or without TGF-β1 for 24 h, cells were fixed by 4% paraformaldehyde and then blocked by QuickBlock™ Blocking Buffer for Immunol Staining (Beyotime). Afterward, the cells were incubated by DYKDDDDK tag Monoclonal antibody (Binds to FLAG^®^ tag epitope) (Proteintech, 66008-4-Ig) and HA tag Polyclonal antibody (Proteintech, 51064-2-AP) overnight. Alexa Flour 647 conjugated antibody and Alexa Flour 488 conjugated antibody were employed to visualize the proteins. Images were acquired with a confocal microscope (CLSM, LSM700, Zeiss, Germany).

## Proximity ligation assay

Duolink assay Duolink^®^ proximity ligation assay (PLA^®^) (Sigma-Aldrich; DUO92101) was performed according to the manufacturer’s instructions. In brief, NRCFs were treated with or without TGF-β1 for 24 h. After fixation and blocking, cells were incubated with PFKFB3-specific antibody (Proteintech, 13763–1-AP) and anti‑OTUD4 antibody (LSBio, LS-C669427), washed with PBS, applied with PLA minus and plus probes, continued with ligation and amplification. Images were acquired with a confocal microscope (CLSM, LSM700, Zeiss).

## Surface plasmon resonance (SPR) analysis

The binding affinity between PFKFB3 and OTUD4 was assayed by a Biacore T200 (GE Healthcare). OTUD4 human recombinant protein (Origene Technologies, Inc., Rockville, MD, USA) was captured on a CM5 chip by a typical amine coupling procedure. Binding sensorgrams were recorded by injecting various concentrations of PFKFB3 solution (Origene) over the immobilized OTUD4 surface. The data were fitted and analyzed to obtain the equilibrium dissociation constant (KD) value.

## Statistical analysis

The data were expressed as mean ± SD. All experiments were performed at least four times. Significant differences were analyzed by one-way ANOVA followed by Bonferroni correction using GraphPad Prism 7. *p*-value < 0.05 was considered statistically significant.

## Results

### Glycolysis was augmented in cardiac fibroblasts upon TGF-β1 stimulus

**Fig. 1:**
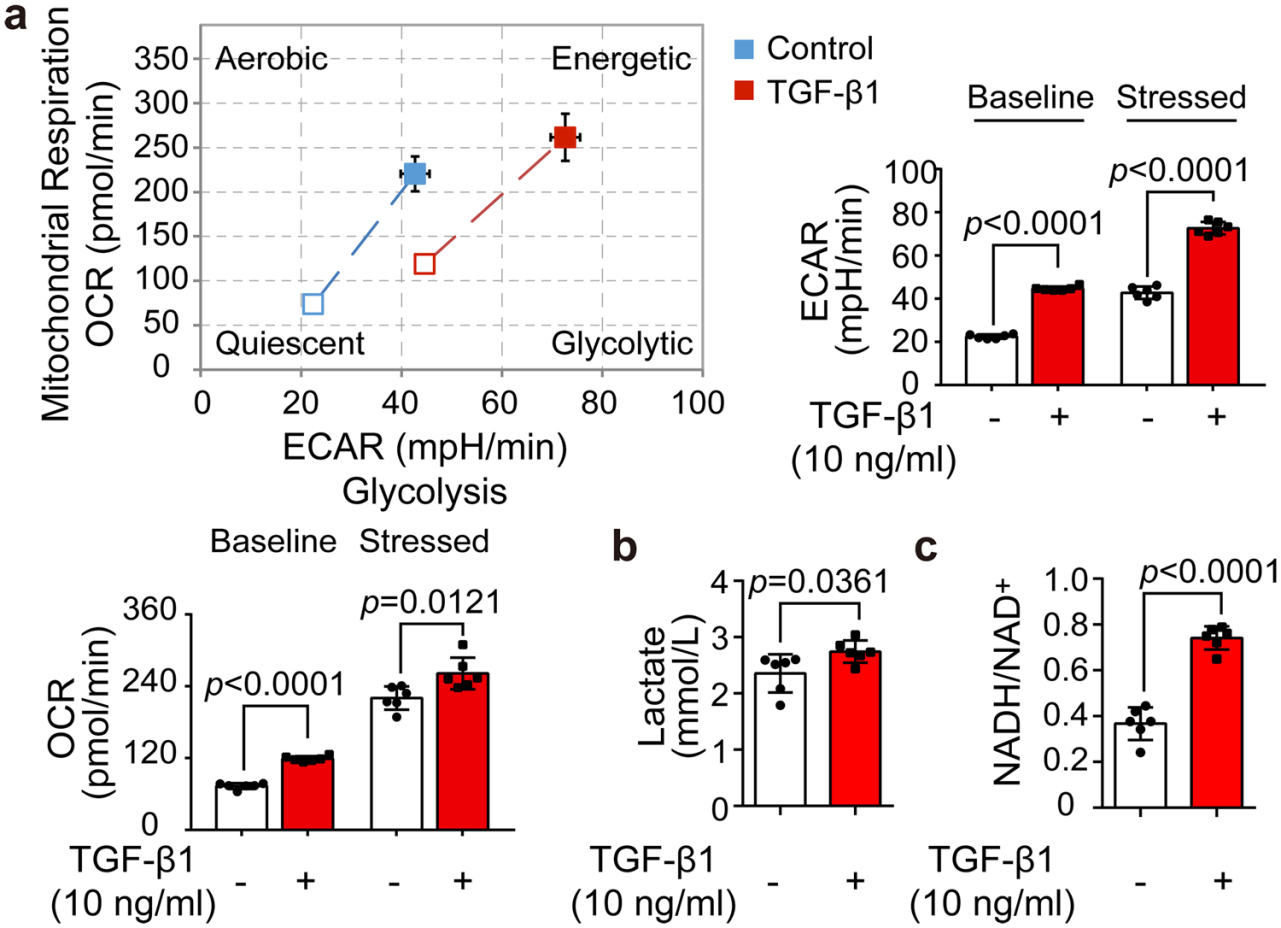
Glycolysis was augmented in cardiac fibroblasts upon TGF-β1 stimulus. **a** Energy phenotypes, mitochondrial oxygen consumption rate, and extracellular acidification rate of neonatal rat cardiac fibroblasts stimulated by TGF-β1 (10 ng/ml, 24 h) assayed in quiescent and stressed conditions (*n* = 6). **b** Lactate levels in cultural supernatant of neonatal rat cardiac fibroblasts subjected to TGF-β1 (10 ng/ml, 24 h) (*n* = 6). **c** NADH/NAD^+^ ratio in neonatal rat cardiac fibroblasts subjected to TGF-β1 (10 ng/ml, 24 h) (*n* = 6). Data were expressed as mean ± SD. ECAR, extracellular acidification rate; OCR, oxygen consumption rate; NAD, nicotinamide adenine dinucleotide; TGF-β1, transform growth factor β1

To investigate if the pathogenesis of cardiac fibrosis involves metabolic reprogramming, we determined the energy phenotype profile of NRCFs differentiated with fibrotic activator TGF-β1. Seahorse extracellular flux analyzer showed that TGF-β1 stimulation led to a significant ECAR increase, an indicator of glycolysis, both in baseline and stressed phases. OCR, a representation of mitochondrial oxidative phosphorylation, is also elevated in response to TGF-β1 (Fig. 1a). Meanwhile, TGF-β1 stimulation increased lactate accumulation (Fig. 1b), a metabolic product of glycolysis, and elevated cytosolic NADH/NAD^+^ ratio (Fig. 1c).

### Glycolytic enzyme PFKFB3 was elevated in fibrotic cardiac fibroblasts

**Fig. 2:**
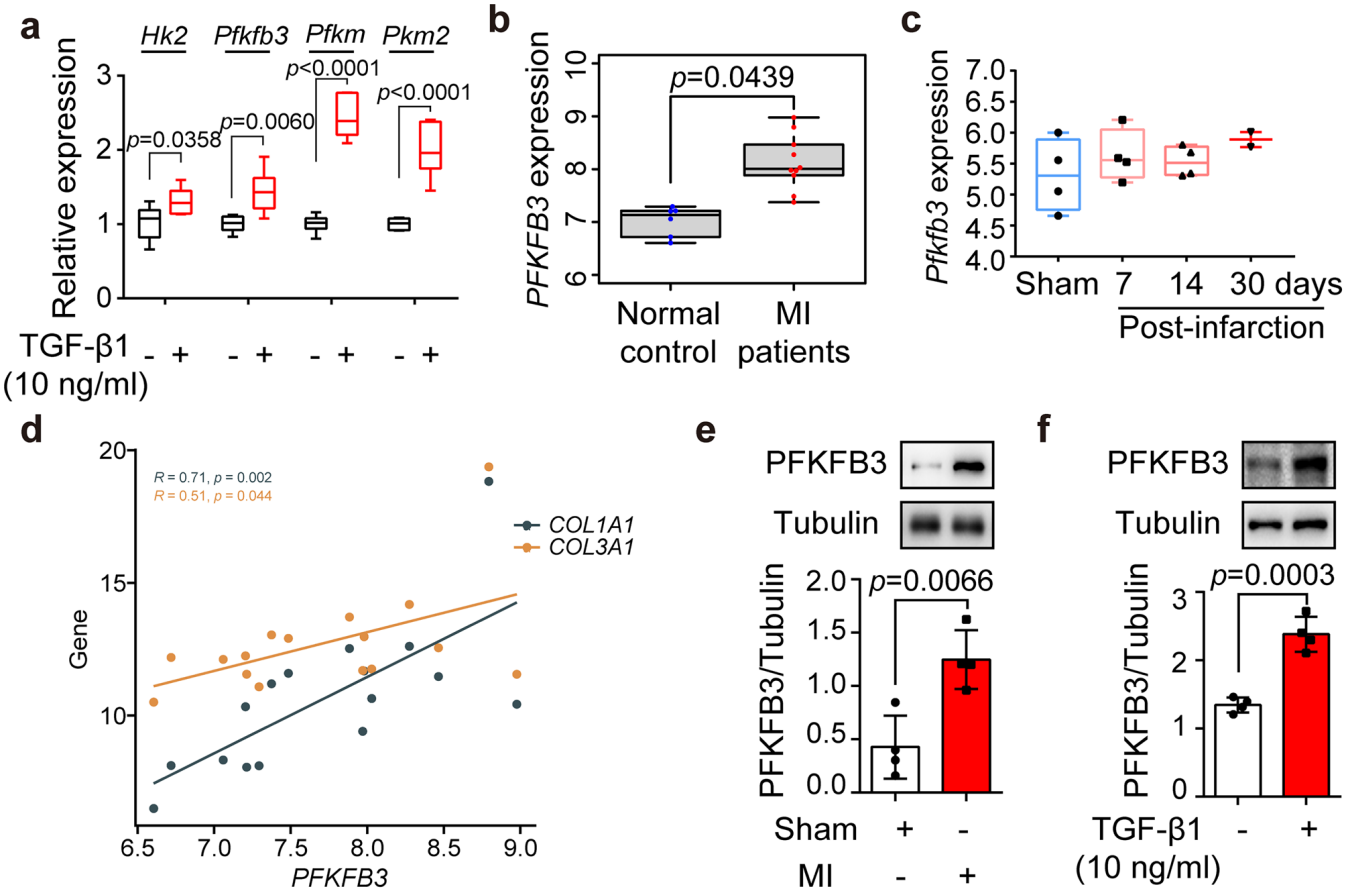
Glycolytic enzyme PFKFB3 was elevated in fibrotic cardiac fibroblasts. **a** mRNA expressions of *Hk2*, *Pfkfb3*, *Pfkm*, and *Pkm* in neonatal rat cardiac fibroblasts treated with TGF-β1 (10 ng/ml, 24 h) (*n* = 4). **b** *PFKFB3* expressions in human cardiac tissue from ischemic patients analyzed based on a publicly available database (GSE132143). **c** *Pfkfb3* expressions in cardiac fibroblasts from mice post-infarction analyzed based on a publicly available database (GSE132143). **d** Analysis of the correlation between *PFKFB3* expression and the levels of fibrotic markers (*COL1A1*; *COL3A1*) based on a publicly available database (GSE132143). **e** PFKFB3 protein expression in primary adult cardiac fibroblasts isolated from mice 28 days post-myocardial infarction (*n* = 4). **f** PFKFB3 protein expression in neonatal rat cardiac fibroblasts subjected to TGF-β1 (10 ng/ml, 24 h) (*n* = 4). Data were expressed as mean ± SD. MI, myocardial infarction

We investigated several glycolytic enzymes in TGF-β1-induced NRCFs that might contribute to the energy phenotype changes. The mRNA expressions of *Hk2*, *Pfkfb3*, *Pfkm*, and *Pkm2* were significantly increased (Fig. 2a). Considering that direct inhibition of key rate-limiting glycolytic enzymes (HK, PFK1, PKM2) may cause unfavorable effects, we focused on PFKFB3, a key modulator of glycolysis. RNA sequencing database (GSE132143) of human cardiac tissue from ischemic patients confirmed that *PFKFB3* expression was up-regulated (Fig. 2b). Consistently, single-cell RNA sequencing database also demonstrated that *Pfkfb3* expression exhibited an increasing trend in the cardiac fibroblasts from mice 7, 14, and 30 days post infarction (Fig. 2c). Additionally, there was a positive correlation between the *Pfkfb3* and fibrotic markers (Fig. 2d). In line with these results, our experiments showed that PFKFB3 exhibited a low basal level of protein expression but was strongly increased in primary adult cardiac fibroblasts isolated from mice 4 weeks post-MI (Fig. 2e, Supplemental Fig. S1a), but not in cardiomyocytes or infiltrating macrophages (Supplemental Fig. S1b, c). Consistently, PFKFB3 was also elevated in NRCFs subjected to TGF-β1 (Fig. 2f).

### Inhibition of PFKFB3-involved glycolysis alleviated the fibrotic phenotypes in TGF-β1-induced cardiac fibroblasts

**Fig. 3:**
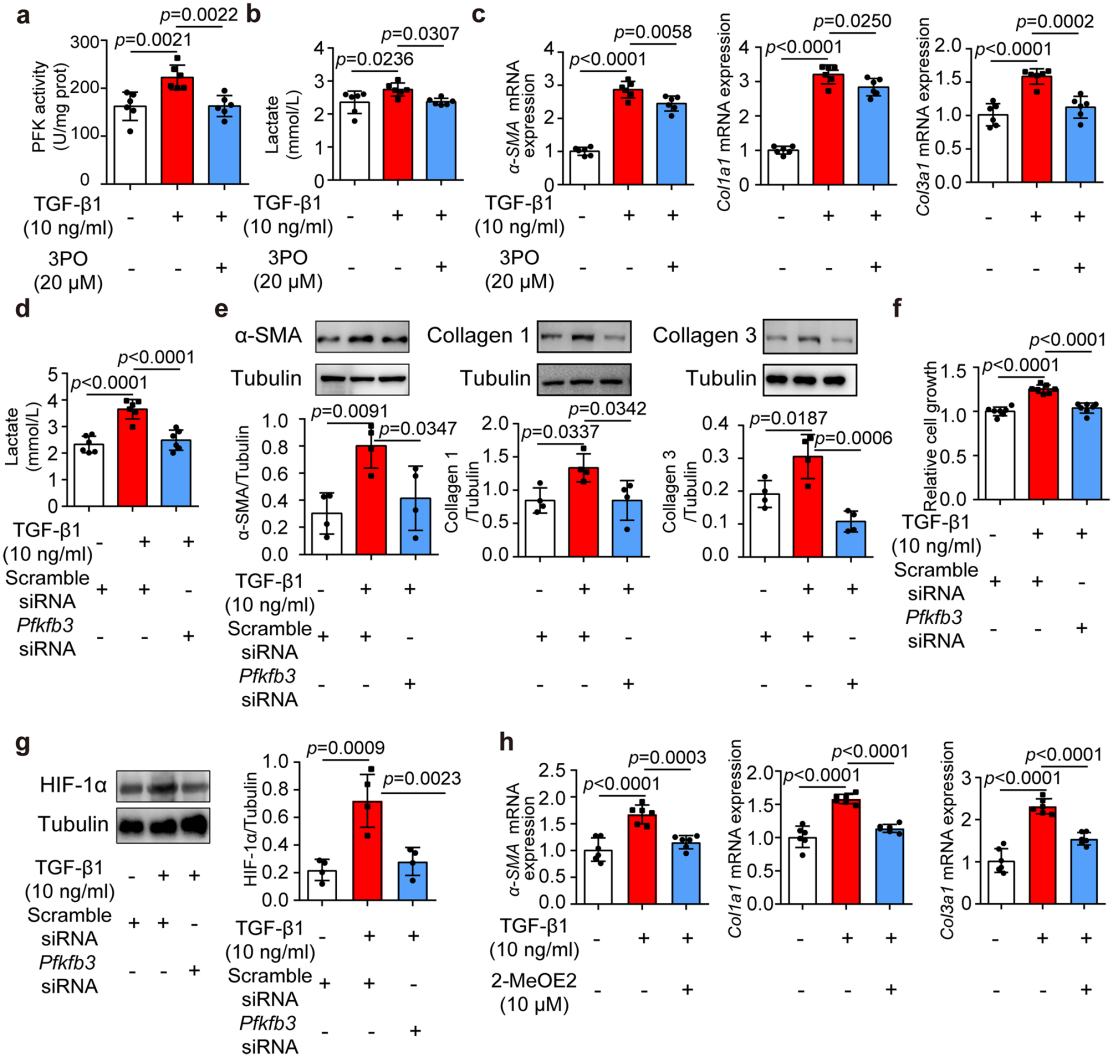
Inhibition of PFKFB3-involved glycolysis alleviated the fibrotic phenotypes in TGF-β1-induced cardiac fibroblasts. **a** Phosphofructokinase activity stimulated with TGF-β1 (10 ng/ml, 24 h) in the presence of 3PO (20 μM, 24 h) (*n* = 6). **b** Lactate levels in cultural supernatant of neonatal rat cardiac fibroblasts subjected to TGF-β1 (10 ng/ml, 24 h) with or without 3PO (20 μM, 24 h) (*n* = 6). **c** mRNA expressions of *α-SMA, Col1a1, Col3a1* in neonatal rat cardiac fibroblasts stimulated by TGF-β1 (10 ng/ml) in the presence or absence of 3PO (20 μM) for 24 h (*n* = 6). **d** Lactate levels in cultural supernatant of neonatal rat cardiac fibroblasts transfected with scramble siRNA or *Pfkfb3* siRNA (*n* = 6). **e** Protein expressions of α-SMA, collagen 1, and collagen 3 in neonatal rat cardiac fibroblasts transfected with scramble siRNA or *Pfkfb3* siRNA (*n* = 4). **f** Cell growth of neonatal rat cardiac fibroblasts incubated with TGF-β1 (10 ng/ml, 24 h) transfected with scramble siRNA or *Pfkfb3* siRNA (*n* = 6). **g** HIF-1α protein level in TGF-β1-treated neonatal rat cardiac fibroblasts transfected with *Pfkfb3* siRNA (*n* = 4). **h** mRNA expressions of *α-SMA, Col1a1, Col3a1* in TGF-β1-treated neonatal rat cardiac fibroblasts treated with HIF-1α inhibitor 2-MeOE2 (*n* = 6). Data were expressed as mean ± SD. HIF-1α, hypoxia-inducible factor 1α; PFK, phosphofructokinase

To investigate the effect of augmented glycolysis on the activation and differentiation of cardiac fibroblasts, we used a specific PFKFB3 inhibitor 3PO to attenuate glycolysis at the given concentration without any cytotoxic effects on cardiomyocytes (Supplemental Fig. S2a). 3PO treatment impaired PFKFB3 enzyme activity, reflected by the activity of phosphofructokinase (Fig. 3a) and reduced extracellular lactate level (Fig. 3b) in TGF-β1-stimulated NRCFs. Importantly, 3PO diminished the TGF-β1-stimulated *α-SMA* gene expression (Fig. 3c), a marker of cardiac fibroblast activation. 3PO was also able to reverse the differentiated phenotypes in mRNAs of *Col1a1* and *Col3a1* of NRCFs (Fig. 3c).

Furthermore, siRNA transfection was employed to silence *Pfkfb3* in NRCFs (Supplemental Fig. S2b)*.* As expected, *Pfkfb3* silencing decreased the extracellular lactate levels in response to TGF-β1 stimulation (Fig. 3d). SiRNA silencing also inhibited the protein expressions of α-SMA, collagen 1, collagen 3 (Fig. 3e), and suppressed proliferation of NRCFs upon TGF-β1 insult (Fig. 3f). In addition, the protein level of hypoxia-inducible factor 1α (HIF-1α) was elevated upon TGF-β1 insult, and this alteration was reversed by *Pfkfb3* silencing (Fig. 3g). Moreover, HIF-1α inhibitor 2-MeOE2 attenuated the increase of *α-SMA*, *Col1a1*, and *Col3a1* expression in TGF-β1-treated cardiac fibroblasts (Fig. 3h), revealing that HIF-1α may function downstream of augmented glycolysis in regulating cardiac fibroblast activation and differentiation.

### Downregulation of PFKFB3 attenuated post-MI cardiac fibrosis in mice

**Fig. 4:**
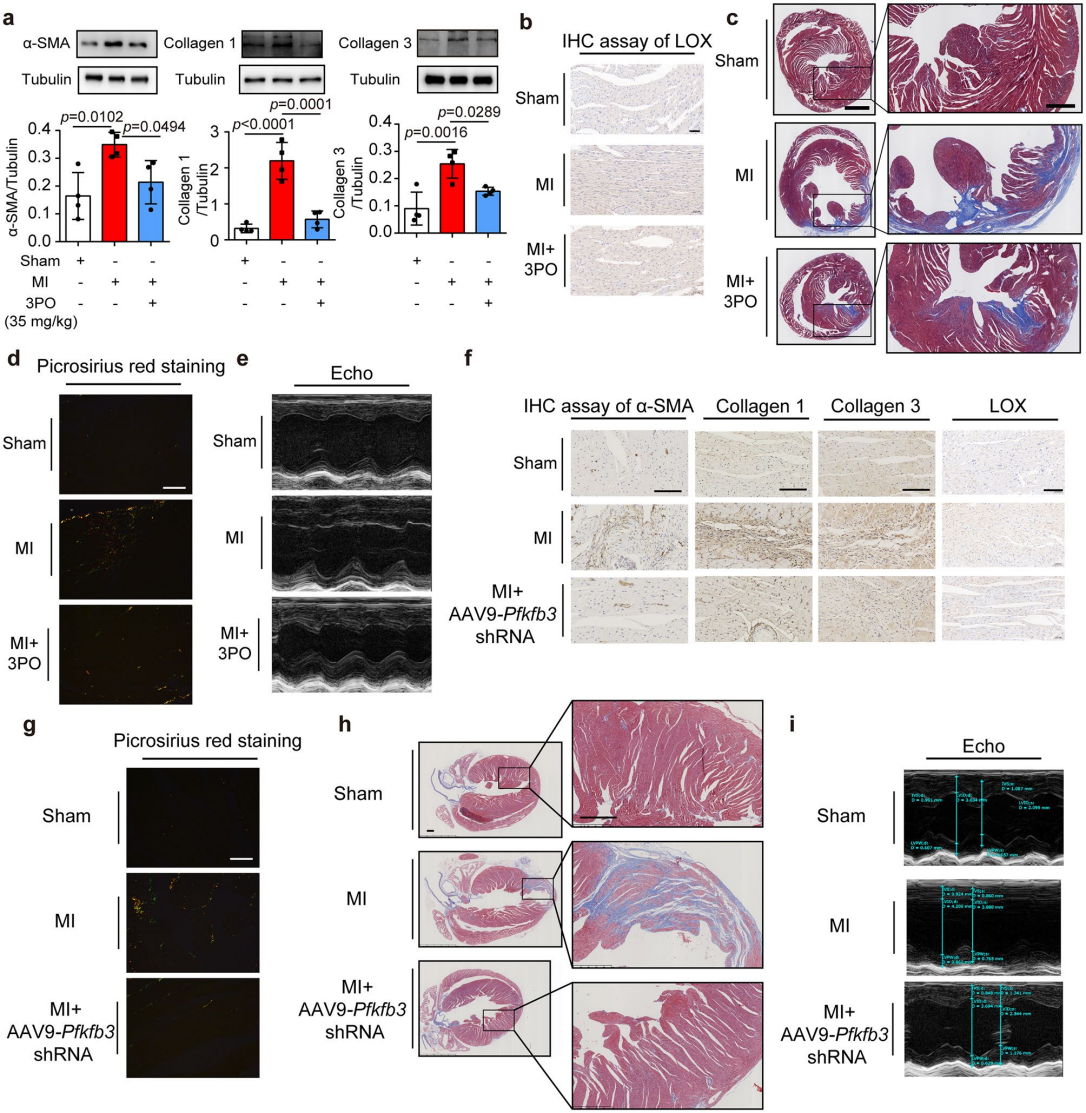
Downregulation of PFKFB3 attenuated post-MI cardiac fibrosis in mice. **a**–**e** Protein expression of α-SMA, collagen type 1, collagen type 3 (**a**, *n* = 4), LOX expression (**b**, scar bar: 50 μm, n = 4), Masson’s staining (**c**, scar bar: 1 mm (left) and 500 µm (right), *n* = 4), picrosirius red staining viewed under polarized light (**d**, scar bar: 50 μm, *n* = 4) and representative M‐mode images by echocardiography (**e**, *n* = 6) of the hearts from mice 28 days post-myocardial infarction with 3PO administration. **f**–**i** The mice were injected with AAV9-*Pfkfb3* shRNA or AAV9-NC prior to LAD ligation surgery. The levels of α-SMA, collagen type 1, collagen type 3 and LOX (**f**, bar: 100 μm, *n* = 4), picrosirius red staining imaged under polarized light (**g**, scar bar: 50 μm, *n* = 4), Masson’s staining (**h**, scar bar: 500 μm, *n* = 4), and representative M‐mode images by echocardiography (**i**, n = 6) of the hearts were assayed 4 weeks post-MI. Data were expressed as mean ± SD. MI, myocardial infarction

The roles of PFKFB3 and glycolysis in cardiac fibrosis were investigated in post-MI mice. Intraperitoneal injection of PFKFB3 inhibitor 3PO (35 mg/kg) inhibited the expressions of α-SMA, collagen 1, and collagen 3 in post-MI mice (Fig. 4a). LOX expression was markedly increased, and this alteration was attenuated by 3PO treatment (Fig. 4b). Masson’s trichrome staining showed that 3PO treatment suppressed cardiac fibrosis (Fig. 4c). A similar result was observed with Picrosirius red staining of collagen contents under polarized light (Fig. 4d). 3PO treatment also improved cardiac functions in ejection fraction (EF%) and fractional shortening (FS%) (Fig. 4e and Table 1).

**Table 1:** Echocardiographic parameters

Parameter	Sham	MI	MI + 3PO	Sham + AAV9-NC	MI + AAV9-NC	MI + AAV9-*Pfkfb3 s*hRNA
LV ejection fraction (%)	62.10 ± 3.48	36.24 ± 7.57^a^	57.58 ± 10.15^b^	60.55 ± 2.73	44.8 ± 2.81^c^	61.33 ± 2.42^d^
LV fraction shortening (%)	32.88 ± 2.63	17.19 ± 3.99^a^	30.11 ± 6.99^b^	31.62 ± 1.84	21.82 ± 1.48^c^	32.33 ± 1.81^d^
LV mass (mg)	72.33 ± 20.56	81.68 ± 23.33	86.36 ± 16.77	76.02 ± 19.92	94.11 ± 6.69	91.25 ± 16.29
LV diastolic volume (μL)	60.98 ± 13.89	65.96 ± 13.10	57.16 ± 12.31	54.03 ± 13.34	64.28 ± 11.81	61.54 ± 8.20
LV systolic volume (μL)	22.88 ± 4.60	42.14 ± 10.21^a^	24.59 ± 9.81^b^	21.46 ± 6.09	35.77 ± 7.72^c^	23.68 ± 2.50^d^
LV internal dimension (diastole, mm)	3.75 ± 0.36	3.88 ± 0.34	3.66 ± 0.31	3.56 ± 0.38	3.84 ± 0.32	3.78 ± 0.20
LV internal dimension (systole, mm)	2.51 ± 0.20	3.21 ± 0.33^a^	2.56 ± 0.38^b^	2.44 ± 0.29	3.01 ± 0.30^c^	2.56 ± 0.11^d^
Interventricular septal dimension (diastole, mm)	0.69 ± 0.14	0.75 ± 0.09	0.92 ± 0.14	0.84 ± 0.14	0.93 ± 0.15	0.91 ± 0.15
Interventricular septal dimension (systole, mm)	1.00 ± 0.20	0.88 ± 0.09	1.22 ± 0.09^b^	1.14 ± 0.10	1.15 ± 0.08	1.34 ± 0.12^d^
LV posterior wall dimension (diastole, mm)	0.71 ± 0.24	0.72 ± 0.15	0.75 ± 0.13	0.73 ± 0.22	0.76 ± 0.13	0.76 ± 0.12
LV posterior wall dimension (systole, mm)	1.08 ± 0.19	0.90 ± 0.25	1.10 ± 0.15	0.91 ± 0.14	0.84 ± 0.13	1.06 ± 0.13^d^

*LV* left ventricular, *MI* myocardial infarction

^a^ *p* < 0.05 vs. Sham

^b^*p* < 0.05 vs. MI

^c^*p* < 0.05 vs. sham + AAV9-NC

^d^*p* < 0.05 vs. MI + AAV9-NC

Furthermore, we silenced cardiac PFKFB3 in mice by tail vein injection of AAV9-*Pfkfb3* shRNA (Supplemental Fig. S3a). The increased levels of α-SMA, collagen 1, collagen 3, and LOX expression following MI were attenuated via* Pfkfb3* knockdown (Fig. 4f). Consistently, silencing *Pfkfb3* decreased collagen accumulation and attenuated cardiac fibrosis (Fig. 4g, h) and preserved cardiac functions of post-MI mice (Fig. 4i and Table 1).

### TGF-β1 treatment reduced the ubiquitination-mediated degradation of PFKFB3

**Fig. 5:**
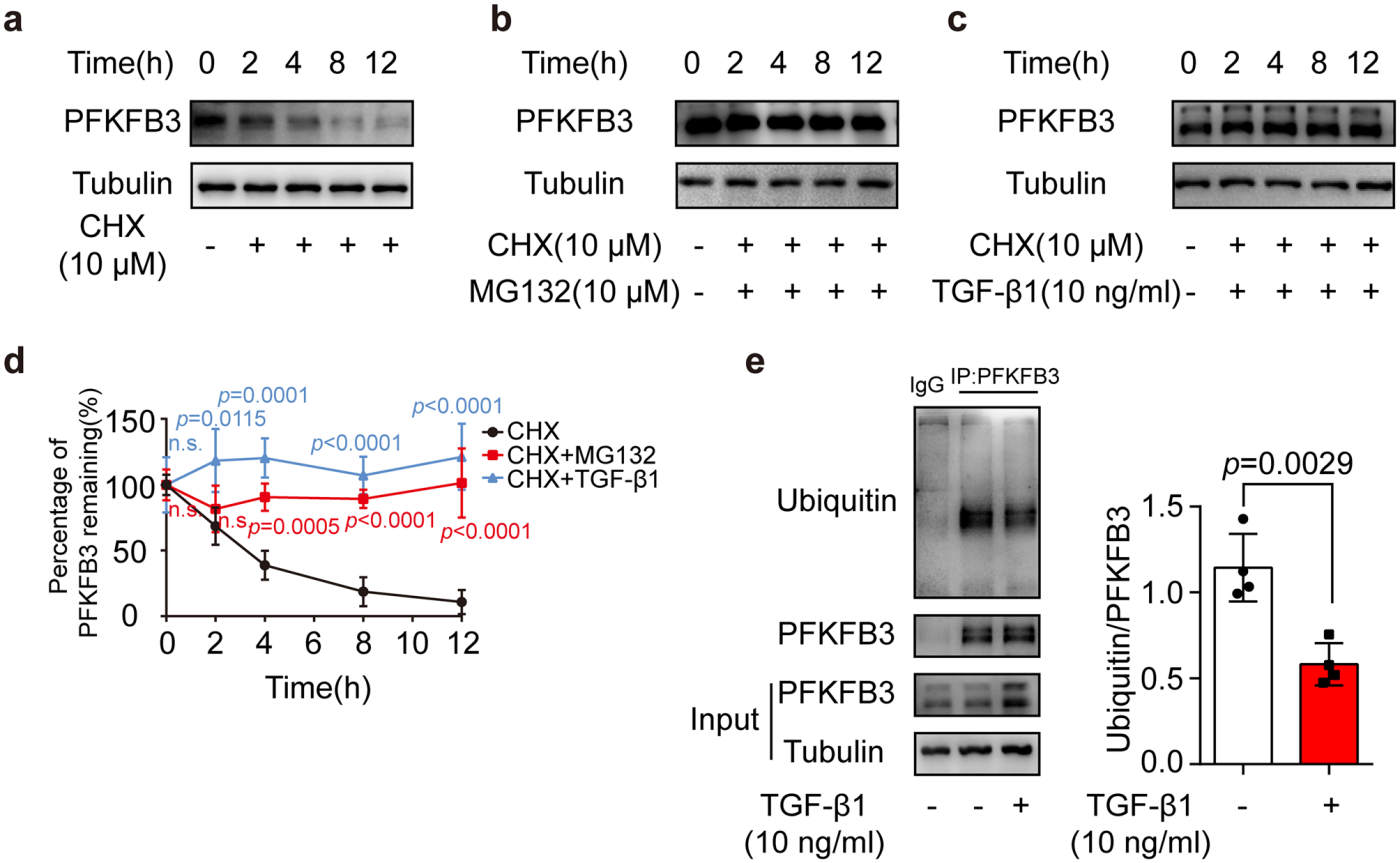
TGF-β1 treatment reduced the ubiquitination-mediated degradation of PFKFB3. **a** Degradation of PFKFB3 in neonatal rat cardiac fibroblasts treated with cycloheximide (10 μM). **b** Degradation of PFKFB3 in neonatal rat cardiac fibroblasts treated with cycloheximide (10 μM) and MG132 (10 μM). **c** Degradation of PFKFB3 in neonatal rat cardiac fibroblasts treated with cycloheximide (10 μM) and TGF-β1 (10 ng/ml). **d** Comparison of degradation of PFKFB3 in neonatal rat cardiac fibroblasts treated with cycloheximide (10 μM), MG132 (10 μM), or TGF-β1 (10 ng/ml), the level of PFKFB3 in 0 h is normalized as 100% (*n* = 4). **e** Ubiquitylation of PFKFB3 purified with immunoprecipitation from neonatal rat cardiac fibroblasts treated with or without TGF-β1 (10 ng/ml, 24 h) (*n* = 4). Data were expressed as mean ± SD. CHX, cycloheximide

PFKFB3 is subjected to constant proteasomal degradation via ubiquitination [22]. We hypothesized that the upregulation of the PFKFB3 protein level was attributable to the reduction of proteasomal degradation. In the presence of CHX, an inhibitor of protein synthesis, the PFKFB3 protein level was degraded gradually over 0–12 h (Fig. 5a). Addition of a proteasomal inhibitor, MG-132, blocked the degradation of PFKFB3 (Fig. 5b). TGF-β1 also significantly inhibited the time-dependent degradation of PFKFB3 (Fig. 5c, d). We then performed immunoprecipitation assays with an anti-PFKFB3 antibody and detected ubiquitin levels by western blotting. As expected, TGF-β1 treatment decreased the level of ubiquitinated PFKFB3 (Fig. 5e).

### TGF-β1 promoted the interaction of PFKFB3 and deubiquitinase OTUD4

**Fig. 6:**
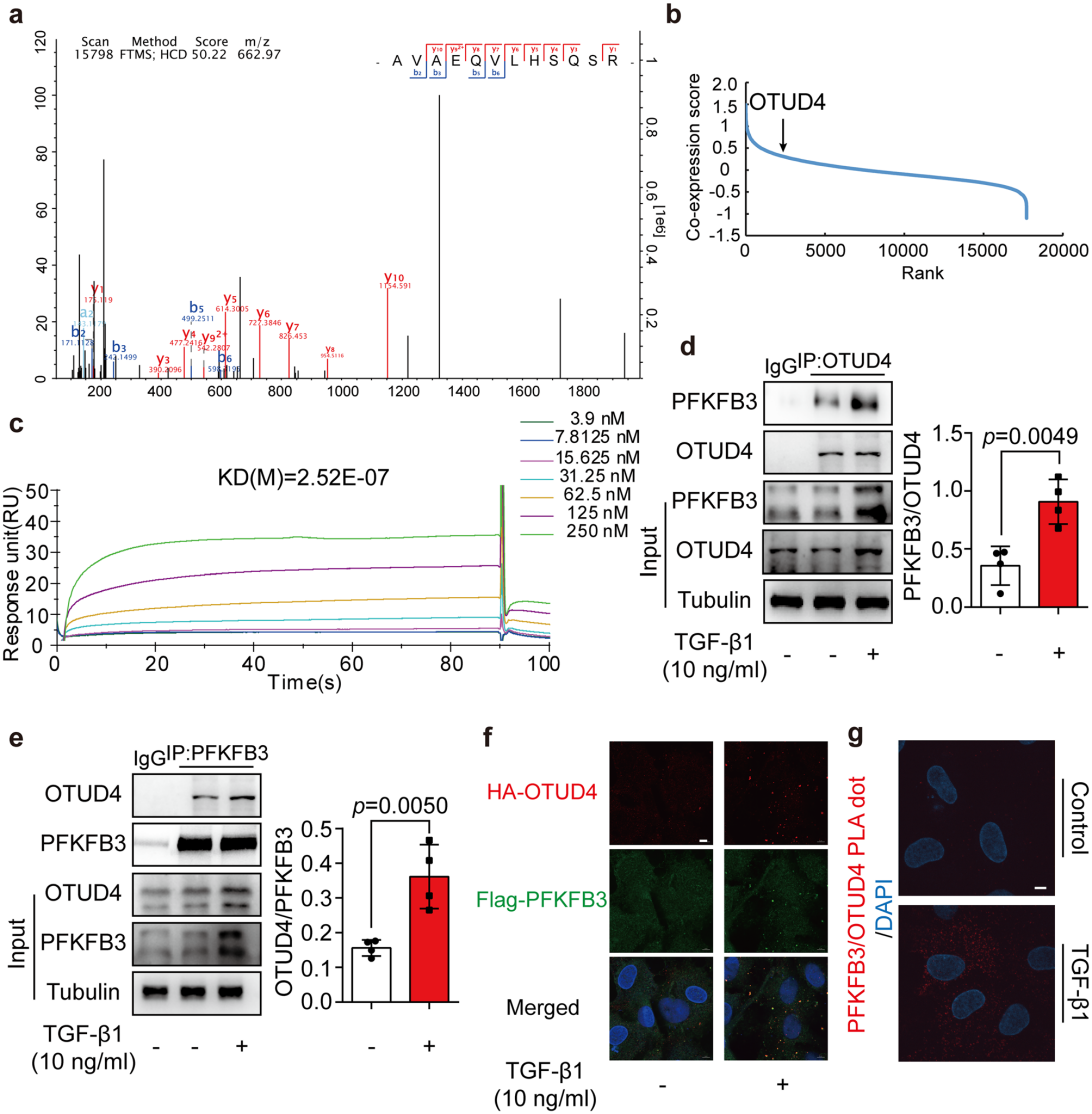
TGF-β1 promoted the interaction of PFKFB3 and deubiquitinase OTUD4. **a** Mass spectrum information of razor peptides to OTUD4 found in immunoprecipitated PFKFB3 complex extracted from TGF-β1 treated neonatal rat cardiac fibroblasts. **b** OTUD4 rank in proteins correlated with PFKFB3 in 39 human organ fibrosis samples from SEEK database. **c** Surface plasma resonance analysis between human recombinant PFKFB3 protein and human recombinant OTUD4 protein. **d** PFKFB3 protein level in precipitated OTUD4 from neonatal rat cardiac fibroblasts incubated with or without TGF-β1 (10 ng/ml, 24 h) (*n* = 4). **e** OTUD4 protein level in precipitated PFKFB3 from neonatal rat cardiac fibroblasts incubated with or without TGF-β1 (10 ng/ml, 24 h) (*n* = 4). **f** Immunofluorescence detection of HA-OTUD4 co-localized with Flag-PFKFB3 in neonatal rat cardiac fibroblasts insulted with TGF-β1 (10 ng/ml, 24 h) (*n* = 4). Scar bar: 5 μm. **g** Proximity ligation assay (PLA) of OTUD4 and PFKFB3 in neonatal rat cardiac fibroblasts incubated with TGF-β1 (10 ng/ml, 24 h) (*n* = 4). Scar bar: 5 μm. Data were expressed as mean ± SD

To clarify the mechanism by which TGF-β1 suppressed PFKFB3 ubiquitylation, we employed Co-IP/MS to identify PFKFB3-binding proteins. We focused on deubiquitinases and found that OTUD4 was a potential interaction protein of PFKFB3 in NRCFs (Fig. 6a, Supplemental Table S3). In 39 human organ fibrosis databases, OTUD4 expression was positively correlated with PFKFB3 in SEEK co-expression database (https://seek.princeton.edu/seek/) (Fig. 6b). More importantly, surface plasmon resonance showed that OTUD4 exhibited a strong binding affinity to PFKFB3 with an estimated equilibrium dissociation constant of 250 nM (Fig. 6c). Co-IP assay demonstrated that TGF-β1 treatment promoted the interaction of endogenous PFKFB3 and OTUD4 (Fig. 6d, e). Consistently, immunofluorescence examination (Fig. 6f) and PLA assay (Fig. 6g) showed the enhanced interaction between OTUD4 and PFKFB3 in TGF-β1 treated NRCFs.

### TGF-β1 promoted PFKFB3 stability dependent on OTUD4

**Fig. 7:**
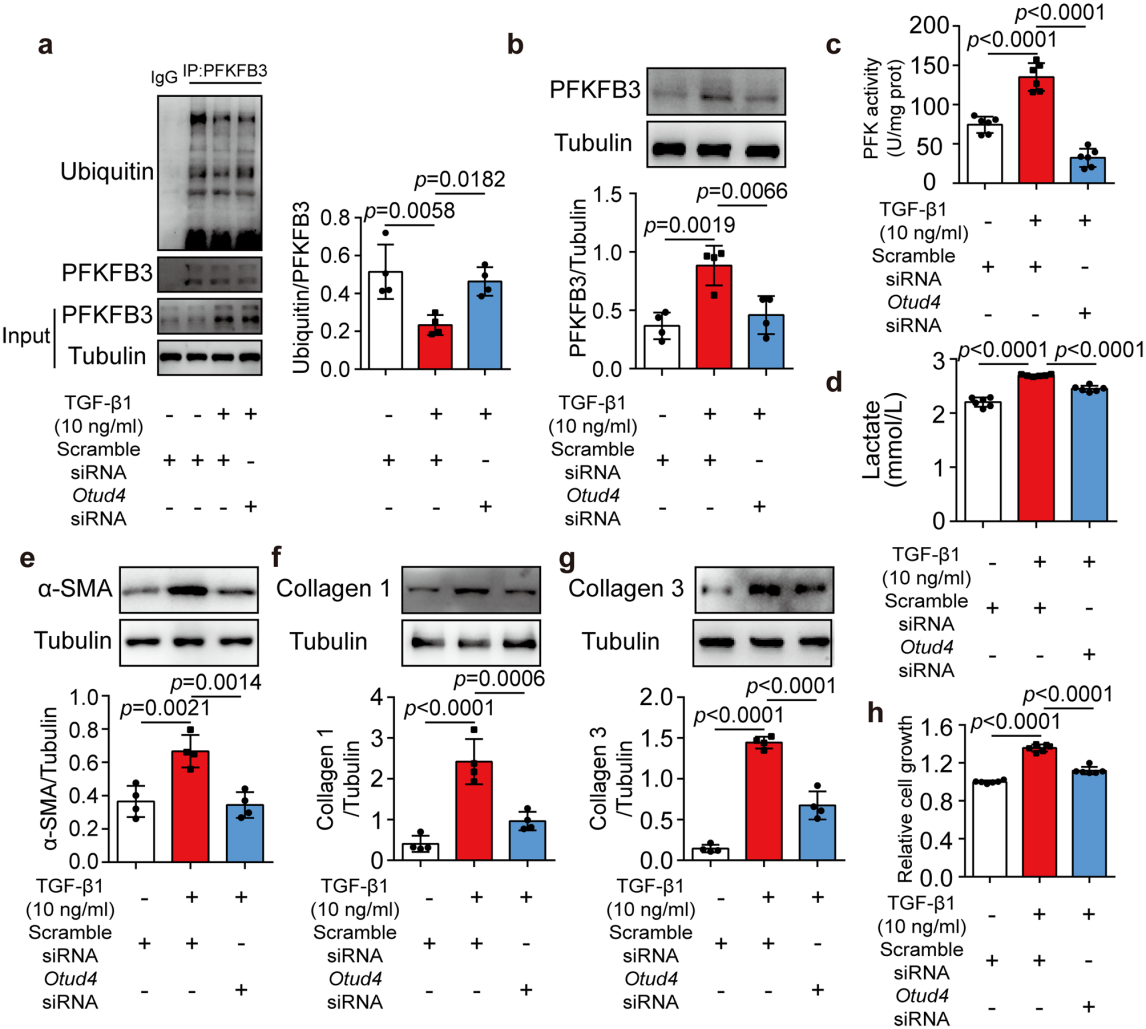
TGF-β1 promoted PFKFB3 stability dependent on OTUD4. **a** Ubiquitylation of PFKFB3 purified with immunoprecipitation from neonatal rat cardiac fibroblasts transfected with scramble siRNA or *Otud4* siRNA and treated with or without TGF-β1 (10 ng/ml, 24 h) (*n* = 4). **b** PFKFB3 protein expression in neonatal rat cardiac fibroblasts transfected with scramble siRNA or *Otud4* siRNA and treated with or without TGF-β1 (10 ng/ml, 24 h) (*n* = 4). **c** and **d**, Phosphofructokinase activity (**c**, *n* = 6), and lactate release (**d**, *n* = 6) of neonatal rat cardiac fibroblasts transfected with *Otud4* siRNA in response to TGF-β1 (10 ng/ml, 24 h). **e**–**h**, Protein expressions of α-SMA (**e**, *n* = 4), collagen 1 (**f**, *n* = 4), collagen 3 (**g**, *n* = 4), and cell growth (**h**, *n* = 6) in neonatal rat cardiac fibroblasts transfected with *Otud4* siRNA upon TGF-β1 insult. Data were expressed as mean ± SD

To further clarify the role of OTUD4 on PFKFB3 stability and fibrotic phenotypes, we knocked down OTUD4 in NRCFs with siRNA (Supplemental Fig. S4a). Results showed that TGF-β1 treatment could reduce the ubiquitination level of PFKFB3, and this process was blocked by OTUD4 knockdown (Fig. 7a). Similarly, up-regulated PFKFB3 protein level and enzyme activity induced by TGF-β1 were reversed when OTUD4 was knocked down (Fig. 7b and c). OTUD4 knockdown also reduced lactate production (Fig. 7d). In agreement, OTUD4 knockdown inhibited the expressions of α-SMA, collagen 1, and collagen 3 and the proliferation of NRCFs upon TGF-β1 insult (Fig. 7e–h).

## Discussion and conclusion

In the present study, we found that the TGF-β1-induced cardiac fibroblast activation and differentiation were associated with PFKFB3-driven glycolysis. Pharmacological and genetic inhibition of PFKFB3 decreased TGF-β1-induced profibrotic phenotypes in NRCFs and attenuated ischemia-induced cardiac fibrosis in mice. We further identified the deubiquitinase OTUD4 as a new binding protein of PFKFB3. TGF-β1 treatment blunted PFKFB3 degradation via OTUD4-mediated deubiquitylation to maintain a high glycolytic phenotype in NRCFs. Our work highlighted that targeting glycolysis-based therapy via inhibition of PFKFB3 served as an alternative way to mitigate cardiac fibrosis.

Cardiac fibrosis occurs in the end stage of MI and impairs cardiac function. TGF-β1 is one of the most important fibrogenic growth factors in cardiac fibrosis. Targeting TGF-β1 is considered an approved therapy for alleviating fibrosis [25]. However, TGF-β inhibition incurred higher mortality and left ventricular dilatation in post-MI mice [26]. Consistently, targeting small mothers against decapentaplegic 3 (SMAD3), the downstream pathway of TGF-β, increased the myofibroblast density [27]. This evidence corroborates that direct inhibiting TGF-β/SMAD signaling leads to more harm than good. As the heart is an energy-intensive organ, perturbations in cardiac energy metabolism are associated with several cardiovascular diseases, such as heart failure and ischemic heart disease [28]. TGF-β1 is also proposed as a metabolic regulator [11]. Hence, we focused on metabolic alternation in cardiac fibrosis. We observed that elevated glycolysis was a crucial character in TGF-β1-stimulated cardiac fibroblasts, in line with the previous reports in several fibrotic diseases [14, 21, 29, 30].

Augmented glycolysis can provide considerable biosynthetic intermediates and energy to support enhanced protein synthesis and rapid proliferation during fibrosis [31]. The metabolic intermediate 3-phosphoglyceric acid contributes to serine and glycine synthesis for collagen production [32]. Lactate, the end-product of glycolysis, can promote collagen hydroxylation [33]. Augmented glycolysis promoted the rise of intermediate succinate to stabilize the transcription factor HIF-1α [14]. In line with this, we found the accumulation of HIF-1α in fibrotic cardiac fibroblasts. Moreover, *Pfkfb3* silencing attenuated the accumulation of HIF-1α upon TGF-β1 insult, suggesting HIF-1α was the downstream of augmented glycolysis. HIF-1α was considered to be required for the activation and differentiation of fibroblasts by binding to the specific responsive element within the *α-SMA* promoter [14]. Consistently, we observed that HIF-1α inhibition attenuated cardiac fibroblast activation and collagen production in TGF-β1-treated cardiac fibroblasts, revealing that HIF-1α-mediated transcription regulation was a critical mechanism through which glycolysis affected cardiac fibrogenesis. Suppressing glycolysis is reported to ameliorate fibrosis in the kidney [29], lung [14], and liver [21]. In line with this, we observed that blocking PFKFB3-involved glycolysis attenuated ECM deposition and fibroblast proliferation to prevent cardiac fibrosis following ischemia.

Glycolytic flux is controlled by the expression or the enzyme activity of key enzymes in the glycolytic pathway. However, direct blockade of glycolysis in the heart may incur some unfavorable effects [18]. 2-deoxy-D-glucose (2-DG), targeting first rate-limiting glycolytic enzyme HK, alleviated post-MI cardiac fibrosis without a reverse of cardiac functions [30]. Being different from the three rate-limiting glycolytic enzymes, PFKFB3 indirectly regulates the glycolytic rate by producing F2,6BP, an allosteric activator of PFK1 [16]. PFKFB3 played a positive role in the normal heart [34]. Elevated PFKFB3 protected cardiomyocytes against hypoxia [35]. However, in long term, PFKFB3-driven aerobic glycolysis accelerated cardiac hypertrophy [35]. In the present study, we found that the overexpression of PFKFB3 in cardiac fibroblasts might be an important feature of cardiac fibrosis post-MI. The PFKFB3 inhibitor 3PO was demonstrated to reduce glycolysis only partially, without causing antiglycolytic effects in normal tissue [14, 21]. It is reported that a mild ramping down of glycolysis by 3PO was able to alleviate lung fibrosis [14]. Similarly, 3PO administration could suppress the development of endothelial-to-mesenchymal transition-associated cardiac fibrosis [36]. In line with these, we observed that pharmacological and genetic inhibition of PFKFB3 attenuated cardiac fibrosis and preserved the cardiac functions indicated by the increased ejection fraction and fraction shortening of post-infarction hearts in mice. Notably, it could not rule out the possibility that the action of 3PO on other cardiac cell types, such as cardiomyocytes and infiltrating macrophages, may also contribute to its in vivo effectiveness. Additionally, systemic injection of AAV-9 shRNA provided highly efficient transduction in the heart, but there was no selectivity in individual cardiac cell types. Therefore, further research on the target depletion of PFKFB3 using cardiac fibroblast-specific knockout mice or AAV-9 vectors carrying myofibroblast-specific promoters would be required.

PFKFB3 level is regulated by multiple aspects, including transcription, translation, post-translational modifications, and protein degradation [20–22, 37, 38]. As reported, TGF-β1 treatment regulates the transcription of *Pfkfb3* mRNA in both cancer cells and fibrotic lung myofibroblasts [14, 39]. Similarly, we observed that in TGF-β1-induced cardiac fibroblasts, the mRNA expression of *Pfkfb3* was increased. Besides the transcriptional regulation, ubiquitin-mediated proteasomal degradation controls the PFKFB3 protein level [22, 40]. Increasing ubiquitination of PFKFB3 promoted its degradation and contributed to inhibiting cell proliferation [40]. Disrupting PFKFB3 stability by accelerating its degradation could inhibit glycolysis to limit the fibrotic process [36]. Consistently, our study showed that TGF-β1 treatment decreased the level of PFKFB3 ubiquitination and stabilized PFKFB3. It is suggested that ubiquitin-mediated degradation is involved in the elevation of PFKFB3 protein levels in activated cardiac fibroblasts.

To further explore the mechanism by which TGF-β1 suppressed PFKFB3 ubiquitination, we found that OTUD4, a deubiquitinase, was a PFKFB3-binding protein and enhanced the stability of PFKFB3. Deubiquitination has attracted increasing attention regarding the regulation of aerobic glycolysis through facilitating protein stability and activity of some glycolytic enzymes [41]. OTUD4, which belongs to the OTU deubiquitinase family. OTU deubiquitinases can block ubiquitin-dependent protein degradation and are involved in various pathways and processes, like profibrotic signaling, proliferation, and glucose metabolism [42–44]. OTUD4 has been reported as a positive regulator of TGF-β signaling [45]. Herein, we showed that TGF-β1 promoted the interaction of OTUD4 and PFKFB3. Through deubiquitination, OTUD4 stabilized the PFKFB3 level and promoted TGF-β1-induced fibroblast activation.

An increased total amount of PFKFB3 facilitates its various post-translational modifications [37, 38]. For example, PFKFB3 can be activated by AMP-activated protein kinase (AMPK)-involved phosphorylation, leading to the increase of glycolytic flux to promote cell proliferation [37, 46]. As a master energy regulator, AMPK activation is known to be involved in the regulation of cardiac fibrosis post-ischemia via diverse mechanisms [47]. As reported, AMPK can activate the TGF-β1 pathway to promote the formation of a mature collagen scar during early post-MI remodeling. However, in adverse post-MI heart remodeling, AMPK activation exerts an anti-fibrotic effect [47]. Additionally, O-GlcNAcylation, as the sensor of oxygen and glucose, is known as a key regulator of glycolytic pathways and is involved in regulating myocardial hypertrophy or fibrosis [48–50]. Evidence showed that O-GlcNAcylation of PFKFB3 was demonstrated to be required for tumor cell proliferation under hypoxia conditions [38]. In line with this, we also observed the increased O-GlcNAcylation of PFKFB3 in fibrotic cardiac fibroblasts (Supplemental Fig. S5a). Further study is required to uncover the role of PFKFB3 or other glycolytic enzymes in regulating cardiac fibrosis post-ischemia from the perspective of post-translational modifications.

In conclusion, we demonstrated that the glycolytic enzyme PFKFB3 was up-regulated to augment glycolysis in fibrotic cardiac fibroblasts. Deubiquitinase OTUD4 bonded to PFKFB3 and enhanced PFKFB3 stability via deubiquitylation upon TGF-β1 treatment. Blockade of PFKFB3 contributed to alleviating fibrotic phenotypes and ameliorating ischemia-induced cardiac fibrosis. Together, our research provided evidence of treating cardiac fibrosis through the regulation of glycolytic reprogramming via targeting PFKFB3.

## Supplementary Information

Below is the link to the electronic supplementary material.

Supplementary file1 (PDF 723 KB)

Supplementary file2 (XLSX 25 KB)

## Data Availability

The authors declare that all data supporting the findings of this study are available within the article or from the corresponding author upon reasonable request.
