# Supplementary material for: Upregulation of glycolytic enzyme PFKFB3 by deubiquitinase OTUD4 promotes cardiac fibrosis post myocardial infarction
Source: J Mol Med (Berl). 2023 May 10;101(6):743–56. doi: 10.1007/s00109-023-02323-6 (PMC10234888; doi:10.1007/s00109-023-02323-6)
Supplement: Supplementary file 1 — Supplementary file1 (PDF 723 KB) [file 109_2023_2323_MOESM1_ESM.pdf]

Upregulation of glycolytic enzyme PFKFB3 by deubiquitinase OTUD4 promotes cardiac fibrosis post myocardial infarction

Feizuo Wang[a,b,1], Xiaojian Yin[b,1], Yuan-Ming Fan[b], Xinyao Zhang[b], Chao Ma[a], Keke Jia[a], Wei Zhou[b], Zongxiang Tang[a]*, Lian-Wen Qi[b]*, Jia Li[a]*

[a]School of Medicine & Holistic Integrative Medicine, Nanjing University of Chinese Medicine, Nanjing, Jiangsu 210023, China.

[b]State Key Laboratory of Natural Medicines, School of Traditional Chinese Pharmacy, China Pharmaceutical University, Nanjing 210009, Jiangsu, China.

***Corresponding to:**

Jia Li, No. 138 Xianlin Avenue, Nanjing 210023, Nanjing University of Chinese Medicine, E-mail address: 460184@njucm.edu.cn.

Lian-Wen Qi, No. 639 Longmian Road, Nanjing 211198, China Pharmaceutical University, E-mail: qilw@cpu.edu.cn.

Zongxiang Tang, No. 138 Xianlin Avenue, Nanjing 210023, Nanjing University of Chinese Medicine, E-mail address: zongxiangtang@njucm.edu.cn.

[1]These authors contributed equally to this work.

Supplemental Methods

Echocardiography

Cardiac function was measured at 4 weeks post-MI by trans-thoracic echocardiography (VisualSonics, Vevo 3100LT). The mice were anesthetized with 3% inhaled isoflurane with O_2 (1 L/min) as a carrier. Then, the isoflurane was reduced to 1-1.5% to maintain a heart rate of 400-450 bpm. The left ventricular (LV) cavity dimensions and wall thickness were measured in at least three beats using M-mode of the long-axis view. The following parameters were measured: LV internal dimension at diastole and systole at the interventricular septal thickness at diastole and systole, left ventricular posterior wall thickness in diastole and systole. LV diastolic and systolic volume, ejection fraction and fractional shortening were calculated with Vevolab software. LV mass was determined by the area-length method.

Primary adult cardiac fibroblasts and cardiomyocytes preparation

Primary adult cardiac fibroblasts and cardiomyocytes were prepared from adult mice 4 weeks post-MI as previously described [1]. Briefly, the chest cavity was opened to expose the still-beating heart. The heart was clamped and perfused with EDTA buffer, perfusion buffer, and collagenase buffer through left ventricular injection. Then, the heart was removed and minced to obtain cell suspension. The cardiomyocytes were separated by gravity, and the cardiac fibroblasts were collected in the supernatant.

Lactate content detection

The lactate level in neonatal rat cardiac fibroblasts (NRCFs) culture supernatant was measured by a lactate assay kit (Jiancheng Corporation Ltd., Nanjing, China). After being treated with or without TGF-β1 for 24 h, the cultural supernatants of NRCFs were collected. Lactate was oxidized by lactate dehydrogenase, and a product of which absorption maximum is at 492nm was generated.

Ratio of NADH/NAD$^+$ quantification

NRCFs were plated in six-well plates. After being insulted by TGF-β1 for 24 h, the cells were processed with NAD$^+$/NADH extraction solution. The content of NAD$^+$ and NADH was measured by the NAD$^+$/NADH Assay Kit with WST-8 (Beyotime Biotechnology, Shanghai, China) following the manufacturer's instructions.

Enzyme activity measurement

Prepared NRCFs were treated with or without TGF-β1 for 24 h. Assay of PFKFB3 activity was performed by a PFK activity assay kit (Beijing Solarbio Science & Technology Co., Ltd., Beijing, China) according to the instruction manuals.

Quantitative real-time PCR

After being treated with or without TGF-β1 for 24 h, the total RNA of NRCFs was isolated by TRIeasyTM Reagent (Yeasen, Shanghai, China). The extracted RNA was reversely transcribed into cDNA. Relative gene expressions were quantified by Hieff™ qPCR SYBR Green Master Mix Kit (Yeasen). The relative mRNA levels were calculated using the $2^{-\Delta\Delta Ct}$ method with reference to β-actin as a housekeeping gene. Primer sequences were presented in Supplemental Table S1.

The information for antibodies in Western blot assay

The information for antibodies was shown as follows: anti-α-tubulin (Beyotime, AF0001); anti-α-Smooth Muscle Actin (Cell signaling technology, 19245); anti-collagen 1 (Proteintech, 14695-1-AP); anti-collagen 3 (Proteintech, 22734-1-AP); anti-PFKFB3 (Proteintech, 13763-1-AP); anti-Ubiquitin (Santa Cruz, sc-8017); anti-OTUD4 (Novus Biologicals, NBP1-36976); anti-HIF-1α (Beyotime, AG2135).

Immunoprecipitation

After treatment, NRCFs were lysed with Pierce IP Lysis Buffer (Thermo Fisher Scientific, Waltham, MA, USA). Cell lysates were incubated with anti-PFKFB3 antibody overnight. Afterward, the lysates were treated with Pierce Protein A/G Magnetic Beads. Bead-bound proteins were eluted for Western blot assay with an anti-ubiquitin antibody or for mass spectrometry analysis.

For the O-GlcNAcylation of PFKFB3 assay, the prepared cell lysates were incubated with O-GlcNAc antibody (Santa sc-59623) followed by Pierce Protein A/G Magnetic Beads treatment to obtain all O-GlcNAc protein. Then, the eluted protein was used for Western blot assay with the anti-PFKFB3 antibody.

References

[1] Ackers-Johnson M, Li PY, Holmes AP, O'Brien SM, Pavlovic D, Foo RS (2016) A Simplified, Langendorff-Free Method for Concomitant Isolation of Viable Cardiac Myocytes and Nonmyocytes From the Adult Mouse Heart. Circ Res 119:909-920. doi: 10.1161/CIRCRESAHA.116.309202

Supplemental Tables

Table S1. Primer sequences quantitative real-time PCR.

Species	Gene	Primer Sequence 5'-3'	
Rat	*Hk2*	Forward:	AGGGTGAGGATGTGGTCA
		Reverse:	TGAGGGTCTTCGTAGCCA
Rat	*Pfkfb3*	Forward:	TGACAAAGGAAGGAGGACAG
		Reverse:	CACAGACGGACTCGATGAAA
Rat	*Pfkm*	Forward:	GGTGCTGAGGAATGAGAAGTG
		Reverse:	CTGTCAAAGGGAGTTGGGTT
Rat	*Pkm*	Forward:	CTGGAGGCTGTTCGCAT
		Reverse:	GGGTCTGTGGATTGACTGG
Rat	*Otud4*	Forward:	GGTGTCTGAAGGTCATGGA
		Reverse:	TAGGCCCAAAGGACTGC
Rat	*α-SMA*	Forward:	GAGTGATGGTTGGAATGG
		Reverse:	GTGATGATGCCGTGTTCT
Rat	*Col1a1*	Forward:	AGAAAAGGGAACCAAAGG
		Reverse:	GGAAGCCAGTCATACCAG
Rat	*Col3a1*	Forward:	GTTTGGAGAATCTATGAATGGTGGC
		Reverse:	GCTGGAAAGAAGTCTGAGGAAGG
Rat	*β-actin*	Forward:	GAGAGGGAAATCGTGCGT
		Reverse:	GGAGGAAGAGGATGCGG

Table S2. The siRNA sequences for cell transfection.

Species	Gene	Sequence 5'-3'	
Rat	*Pfkfb3*	sense:	GCUGCCUACUAGCCUACUUTT
		antisense:	AAGUAGGCUAGUAGGCAGCTT
Rat	*Otud4*	sense:	GCCCAGCAGUCUAUAGAAATT
		antisense:	UUUCUAUAGACUGCUGGGCTT

Table S3. Potential interaction proteins of PFKFB3 identified by Co-IP/MS (Uploaded as a separate Excel file).

Supplemental Figures

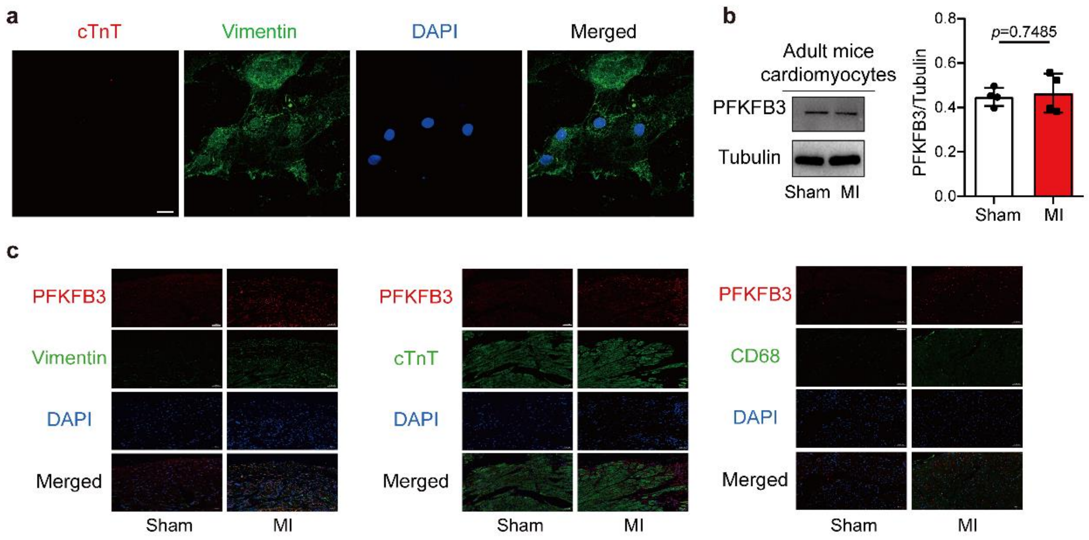

Supple Fig. S1 Primary adult cardiac fibroblasts and cardiomyocytes were prepared from adult mice 4 weeks post-myocardial infarction. a, Primary cultured fibroblasts were identified by the staining of fibroblast marker vimentin. The absence of adhered cardiomyocytes was confirmed by negative cardiac troponin-T staining. Scar bar: 20 μm. b, PFKFB3 protein level in primary adult cardiomyocytes detected by Western blot (n=4). c, Immunofluorescence colocalization of PFKFB3 and different cardiac cell-specific markers in the heart tissue section from post-MI mice. Individual cardiac cell types, including cardiomyocytes, cardiac fibroblasts or infiltrating macrophages, were labeled with cell-specific marker cTNT (Thermo, MA5-12960), vimentin (Proteintech, 60330-1-Ig) or CD68 (Abcam, ab125212) antibodies, respectively. Bar, 50 μm. Data were expressed as mean ± SD.

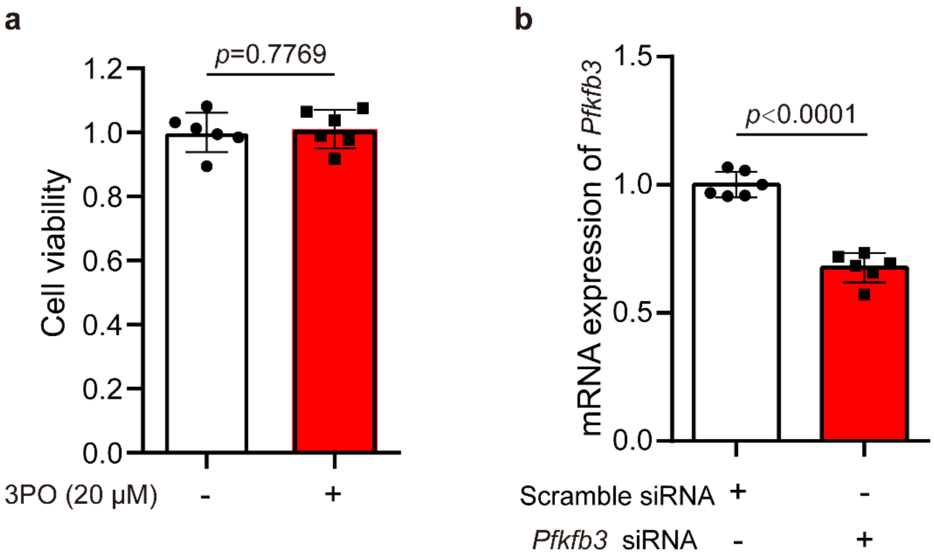

Supple Fig. S2 a, Neonatal rat cardiomyocytes were prepared and treated with 3PO under basal conditions. Cell viability was assayed by a CCK-8 kit (n=6). b, Knockdown efficiency of *Pfkfb3* in neonatal rat cardiac fibroblasts transfected with scramble siRNA or *Pfkfb3* siRNA was determined by real-time quantitative PCR (n=6). Data were expressed as mean ± SD.

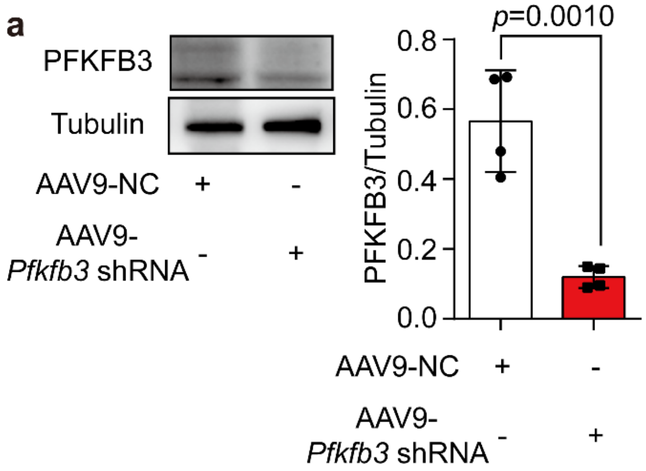

Supple Fig. S3 a, PFKFB3 protein level in heart tissue from mice injected with AAV9-NC or AAV9-*Pfkfb3* shRNA was determined by Western blot (n=4). Data were expressed as mean ± SD.

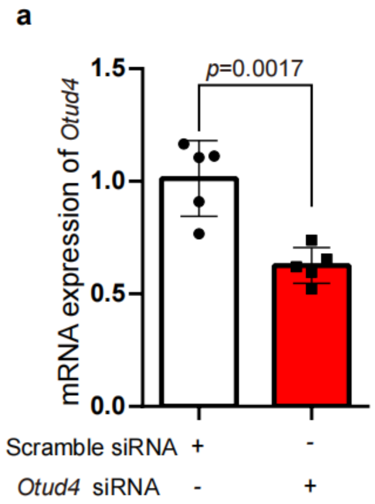

Supple Fig. S4 a, Knockdown efficiency of *Otud4* in neonatal rat cardiac fibroblasts transfected with scramble siRNA or *Otud4* siRNA (n=5). Data were expressed as mean ± SD.

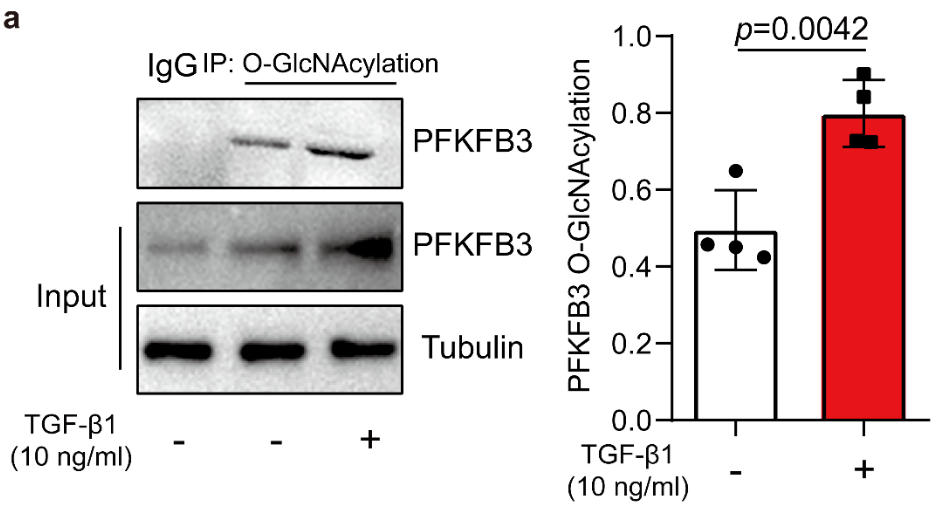

Supple Fig. S5 a, O-GlcNAcylation of PFKFB3 in neonatal rat cardiac fibroblasts treated with or without TGF-β1 (10 ng/ml, 24 h) was detected by immunoprecipitation and Western blot (n=4). Data were expressed as mean ± SD.
